# Changes in health-related quality of life, depression, and fear of progression during oncological inpatient rehabilitation and beyond: a longitudinal study

**DOI:** 10.1007/s00520-024-08800-z

**Published:** 2024-09-02

**Authors:** Jürgen M. Giesler, Joachim Weis

**Affiliations:** 1 Section of Health Care Research and Rehabilitation Research, Institute of Medical Biometry and Statistics, University of Freiburg Medical Center, Hugstetter Str. 49, 79106 Freiburg, Germany; 2 Comprehensive Cancer Center, Department of Self-Help Research, University of Freiburg Medical Center, Freiburg, Germany

**Keywords:** Cancer, Oncological rehabilitation, Health-related quality of life, Depression, Fear of progression, Survivorship

## Abstract

**Purpose:**

Studies evaluating oncological inpatient rehabilitation rarely include follow-up intervals beyond 6 months and larger proportions of patients other than those with breast cancer. Therefore, this study investigated changes in health-related quality of life (HRQoL), depression, and fear of progression of patients with breast, colorectal, or prostate cancer from the beginning to the end of oncological rehabilitation and a 9-month follow-up.

**Methods:**

Three hundred seventy-seven patients with breast, colorectal, or prostate cancer undergoing oncological inpatient rehabilitation (median age 61 years, 49% female) completed the EORTC QLQ-C30, the PHQ-9, and the FoP-Q-SF at each measurement point. Data analysis used 3 (tumor site) × 3 (time of measurement) repeated measures ANCOVAs with patient age and time since diagnosis as covariates. At each time point, we also compared our sample to the general population on the measures used.

**Results:**

Having controlled for the covariates, we found significant effects of tumor site, which were small except for Diarrhea. Effects of time of measurement were often significant and in part at least medium in size indicating improvement of HRQoL and depression during rehabilitation. At follow-up, some HRQoL domains and depression deteriorated. Women with breast cancer, in particular, showed a greater decrease in emotional functioning then. Compared to the general population, the sample’s HRQoL and depression were significantly worse on most occasions.

**Conclusion:**

Oncological inpatient rehabilitation may improve HRQoL. The subsequent and in part differential deterioration in some HRQoL domains suggests a need for further follow-up care within survivorship programs.

## Introduction

The diagnosis of cancer and cancer treatment is frequently associated with acute, long-term, and late sequelae affecting the patient’s physical and psychological functioning, including, e.g., health-related quality of life (HRQoL) [1], emotional distress [2, 3], anxiety and depression [4–6], and fear of progression (FoP) [7]. Aimed at reducing the impact of such disabling and handicapping conditions, oncological rehabilitation attempts to enable patients to adapt to their new situation by regaining physical, social, psychological, and work-related functioning [8]. Its interventions range from techniques targeting specific limitations to more comprehensive multi-modal inpatient approaches. Given current epidemiological trends in cancer incidence and survival, the relevance of cancer rehabilitation with respect to survivorship is expected to even increase in the future [8].

In German-speaking countries, oncological rehabilitation is provided either in an inpatient format at specialized centers over a 3-week period on average or in an outpatient format. In line with the overall goals of oncological rehabilitation, it may include medical treatment, diet counseling, exercise and physical training, health education, psychological support, mind–body interventions, and social counseling tailored to the individual patient’s needs. Addressing key areas of impairment referred to above, studies evaluating the effects of oncological inpatient rehabilitation have frequently included HRQoL, depression, anxiety, or FoP as patient-reported outcomes (PRO) [9–29]. Primarily employing quasi-experimental, longitudinal designs, the majority of these studies [9, 12, 13, 15, 16, 18, 19, 21, 23, 24, 27, 28] included more than two measurement points demonstrating mostly positive and in part stable changes. However, only seven of these studies covered follow-up intervals beyond 6 months after the end of rehabilitation [12, 15, 18, 19, 21, 23, 27]. In addition, even fewer studies included larger proportions of patients with other than breast cancer, like, e.g., colorectal [11, 22, 24, 26] or prostate cancer [15, 23, 24]. Consequently, knowledge on the development of HRQoL, depression, and FoP beyond the end of oncological inpatient rehabilitation is largely scarce as is knowledge on whether patients with different cancers might differ in their development in regard to these outcomes in the long term. Information on these outcomes might, among other things, help identify potential differences in rehabilitation and care needs between different tumor diagnostic groups.

Given these limitations of research in this field of oncological inpatient rehabilitation, the present study aimed to investigate whether.

- HRQoL, depression, and FoP change during oncological inpatient rehabilitation and the subsequent 9 months
- Breast, colorectal, and prostate cancer differ in regard to their potential changes in these PROs
- And to compare the sample’s HRQoL, depression, and FoP to reference values of the general population [30, 31] and to a recommended cut-off for dysfunctional FoP [32]

The study was part of a larger research project focusing upon the measurement of patient competencies in the context of cancer, their potential change during rehabilitation and beyond, and their relationship with HRQoL, depression, and FoP. Results on the first two of these issues have already been reported elsewhere [33, 34].

## Methods

### Design and procedure

The study included *N* = 377 patients from the overall project who received inpatient rehabilitation for breast, colorectal, or prostate cancer in nine clinics for oncological rehabilitation. These cancer types had been chosen to represent three of the most frequent tumor entities. Eligibility criteria further required patients to be at least 18 years old, sufficiently fluent in German, willing to participate in a questionnaire study and not suffering from acute psychiatric illness were eligible for the study. After having been informed about the study by clinic staff and providing written consent, patients completed questionnaires at the beginning and at the end of rehabilitation, and 9 months later. As twenty of the patients had experienced a local recurrence lately, they were excluded from data analyses in order to create a more homogeneous sample in regard to potential treatments received during rehabilitation, resulting in *N* = 357 for analysis.

The study was approved by the University of Freiburg Ethics Committee (No. 359/12) and registered at the German Register of Clinical Studies (No. DRKS00004410).

### Measures

*HRQoL* was measured with the EORTC Quality of Life Questionnaire C-30 (EORTC QLQ-C30) [35]. It includes 30 items covering several domains of functioning and symptoms of cancer patients (global health, physical functioning, fatigue, etc.). Functioning and symptom scale scores represent sums of item sets and are transformed to range from 0 to 100. Higher scores on the global health and functioning scales indicate better quality of life, and higher scores on the symptom scales indicate higher symptom burden. In the present sample, the median internal consistency (Cronbach’s *α*) of the EORTC QLQ-C30 multi-item scales was *Mdn* = 0.85 at baseline.

*Depression* was measured with the German version of the PHQ-9 [36], a module of the Patient Health Questionnaire assessing symptoms of a major depressive disorder by nine items. Summing across item responses gives a scale score ranging from 0 to 27, with higher scores indicating higher depression. In this sample, the internal consistency at baseline was 0.82.

*FoP* was measured with the Fear of Progression Questionnaire short form (*FoP-Q-SF)* [37]. Its 12 items address various cognitive and emotional facets of FoP. Summing across all items yields a score ranging from 12 to 60, with higher scores reflecting higher FoP. A score of 34 or above is assumed to be indicative of a dysfunctional level of FoP [32]. At baseline, the internal consistency in this sample was 0.82.

Selected *sociodemographic data* were provided by patients’ self-reports. *Medical data* were obtained from patients’ medical records at the collaborating centers.

### Statistical methods

Power calculations for the overall project were specified for a 3 × 2 × 3 repeated measures design with the between groups factors of tumor site and curative vs. palliative treatment situation and three measurement points [34]. Consulting appropriate statistical tables [38], a sample size of *N* = 288 appeared adequate for detecting small (*f* = 0.1) interaction effects of these three factors given a type one error of 0.01 and a power (1–*β*) of 0.80. In order to improve the chances of detecting a smaller than medium effect of the tumor site somewhat, we increased the intended cell size per site to 136, totaling *N* = 408. Assuming a dropout of 25%, the overall project aimed to recruit 512 participants at baseline in order to meet this requirement.

Data analysis included descriptive statistics for sociodemographic and medical variables and *χ*^*2*^-tests and one-way ANOVAs in order to determine baseline differences by tumor site. In order to analyze changes across time, we employed a 3 (tumor site: S) × 3 (time of measurement, T) repeated measures analysis of covariance (RM-ANCOVA) design that included patient age and TsD as covariates controlling for respective baseline between groups differences described further below. Testing the effects involving time of measurement, we used Huyn-Feldt-adjusted *F*-values where indicated. Post-hoc between-group comparisons included Bonferroni adjustments. Comparing the sample’s PRO overall means to a respective reference value [30–32], we used two-tailed one-sample *t*-tests. As effect size measures, Cohen’s *d*, *ϕ*, or (partial) *η*^*2*^ were employed. Throughout, missing values were not imputed. Employing *χ*^*2*^-techniques or *t*-tests, we compared patients available at follow-up with dropouts on selected sociodemographic and medical variables and baseline PROs in order to determine potential bias from dropouts.

## Results

### Sample

Patients were 61.1 years old on average (*SD* = 9.4). Mean age varied significantly as a function of tumor site (*F* = 24.0, df: 2, 350; *p* < 0.001, *η*^*2*^ = 0.12; breast cancer: *M* = 56.8, colorectal cancer: *M* = 60.8, and prostate cancer: *M* = 65.2 years). Median TsD was *Mdn* = 9.4 months (range 0.7–325). TsD also varied significantly as a function of the tumor site. It was longer in breast cancer women (*Mdn* = 12.8 months) compared to colorectal and prostate cancer patients (*Mdn* = 8.4 and 4.1 months, respectively; Kruskal–Wallis *H* = 42.3, df: *2*, *p* < 0.001, *η*^*2*^ = 0.13.

Table 1 presents further information on sociodemographic and medical sample baseline characteristics. In most of these, diagnostic groups differ significantly, with effect sizes being mostly small or medium, except for tumor size (T_1_: breast cancer 39%, colorectal cancer 11%, prostate cancer 5%, *ϕ* = 0.52).

**Table 1:** Absolute (f) and relative (%) frequencies of sociodemographic and medical sample characteristics by tumor site

Tumor site	Breast		Colorectal		Prostate		All			
Characteristic^a^	f	%	f	%	f	%	f	%	*χ*^*2*^	*ϕ*
Gender (*n* = 357)									n.c^b^	
Female	98	100	69	49	-	-	167	47		
Male	-	-	72	51	118	100	190	53		
Marital status (*n* = 354)									20.42^**^	.24
Single	12	12	14	10	1	1	27	8		
Married/cohabitating	58	60	94	67	97	84	249	70		
Divorced/separated	18	19	18	13	11	10	47	14		
Widowed	9	9	15	11	7	6	31	9		
Education (*n* = 343)									5.54	-
9 years	24	25	44	33	35	30	103	30		
10 years	35	36	42	31	30	27	107	31		
12 years	11	11	15	11	20	18	46	13		
13 years	27	28	33	25	27	24	87	25		
Employment (*n* = 354)									16.97^***^	.22
Yes (full-time or other)	69	71	81	57	50	43	200	57		
No	28	29	60	43	66	57	154	43		
T (*n* = 318)^c^									86.41^***^	.52
T_1_	31	39	14	11	5	5	50	16		
T_2_	38	48	28	22	50	46	116	37		
T_3_	10	13	70	54	49	45	129	41		
T_4_	1	1	18	14	4	4	23	7		
N (*n* = 335)									35.54^***^	.33
Negative	41	47	69	53	68	59	178	53		
Positive	45	51	61	47	30	26	136	41		
X	2	2	1	1	18	16	21	6		
Primary metastases (*n* = 326)									8.60	-
No	72	87	104	80	93	82	269	83		
Yes	10	12	22	17	11	10	43	13		
X	1	1	4	3	9	8	14	4		
Secondary metastases (*n* = 222)									31.39^***^	.38
No	25	50	65	92	83	82	173	78		
Yes	25	50	6	9	18	18	49	22		

Significant *χ*^2^-values in bold; *ϕ* reported for significant *χ*^2^-values only

^a^*n’s* less than 357 result from missing values in row and/or column variables; ^b^n.c., not computed; ^c^analysis restricted to tumor stages T_1_ through T_4_ in order to avoid expected cell frequencies less than 5 when including T_is_, T_0_, and X

^*^p ≤ .05; **p ≤ 01, ***p ≤ .001

At follow-up, 50 patients (14%) had dropped out of the study. Dropouts and completers were comparable on all PRO baseline measures except for Nausea and Vomiting (completers scoring lower, *p* < 0.05), the effect size being small, however (*η*^*2*^ < 0.05). Sociodemographic and medical characteristics showed only a few significant and small differences between completers and dropouts. Compared to dropouts, a higher proportion of completers received curative treatment and showed no metastases, while tumor stage was either “indeterminate” or T_4_ in a smaller proportion of them (*p* ≤ 0.01, *ϕ* being 0.19, 0.25, and 0.21, respectively).

### Differences between tumor diagnostic groups and changes across time

Table 2 shows few significant and mostly small effects (*η*^*2*^ < 0.06) of the covariates patient age and TsD. The small effect of patient age on social functioning indicates that higher age is associated with better social functioning (*β* = 0.18, not in Table 2), while its moderate effect on financial difficulties implies higher age to go along with fewer difficulties (*β* =  − 0.26). The small significant effects of TsD on fatigue, nausea and vomiting, pain, and constipation indicate a longer TsD to be associated with higher symptom burden (0.14 ≤ *β* ≤ 0.17).

**Table 2:** *F*-values and partial *η*^2^ from 3 (tumor site, S) × 3 (time, T) repeated measurement ANCOVAs with patient age (Age) and TsD as covariates (240 ≤ *n* ≤ 287)

Scale	Covariate, main, and interaction effect terms					
	Statistic	Age	TsD	Site	Time	Time × Site
EORTC QLQ C-30 functioning scales						
Physical functioning	*F*	2.06	1.09	3.74^*^	32.00^***^	2.00
	_*p*_*η*^*2*^	–	–	.03	.10	**–**
Role functioning	*F*	3.01	2.02	.48	45.87^***^	1.40
	_*p*_*η*^*2*^	–	–	–	.14	**–**
Emotional functioning	*F*	.01	1.40	2.16	82.92^***^	5.68***
	_*p*_*η*^*2*^	–	–	–	.23	.04
Cognitive functioning	*F*	2.53	2.69	5.60^**^	17.24^***^	2.28
	_*p*_*η*^*2*^	–	–	.04	.06	–
Social functioning	*F*	8.38**	2.72	.94	55.01^***^	.26
	_*p*_*η*^*2*^	.03	–	–	.17	–
Global health	*F*	.18	2.81	.40	54.95^***^	.20
	_*p*_*η*^*2*^	–	–	–	.17	–
Depression and fear of progression						
Depression	*F*	.64	.73	3.46^*^	57.01^***^	5.66***
	_*p*_*η*^*2*^	–	–	.03	.18	.04
Fear of progression	*F*	1.26	.79	1.16	1.98	.73
	_*p*_*η*^*2*^	–	–	–	–	–
EORTC QLQ C-30 symptom scales						
Fatigue	*F*	.02	7.73^*^	6.34^**^	52.87^***^	.57
	_*p*_*η*^*2*^	–	.03	.04	.16	**–**
Nausea and vomiting	*F*	2.89	4.67^*^	1.25	2.94	1.10
	_*p*_*η*^*2*^	–	.02	–	–	–
Pain	*F*	.05	5.39^*^	4.17^*^	20.74^***^	1.39
	_*p*_*η*^*2*^	–	.02	.03	.07	–
Dyspnea	*F*	.23	1.26	7.81^***^	10.67^***^	3.52^*^
	_*p*_*η*^*2*^	–	–	.05	.04	.03
Insomnia	*F*	.99	.64	4.45^*^	14.68^***^	.57
	_*p*_*η*^*2*^	–	–	.03	.05	–
Appetite loss	*F*	.00	.47	3.44^*^	12.00^***^	2.68*
	_*p*_*η*^*2*^	–	–	.02	.04	.02
Constipation	*F*	3.26	5.97^*^	.54	3.20*	1.06
	_*p*_*η*^*2*^	–	.02	–	.01	–
Diarrhea	*F*	1.26	1.42	25.68^***^	8.40^***^	.60
	_*p*_*η*^*2*^	–	–	.16	.03	–
Financial difficulties	*F*	18.00^***^	.02	6.28^**^	1.20	.90
	_*p*_*η*^*2*^	.06	–	.04	–	–

*Note*: Significant *F*-values in bold

* p ≤ .05; ** p ≤ 01, *** p ≤ .001

Having controlled for both covariates, we find several significant effects of tumor site, which are large for diarrhea and small for physical functioning, cognitive functioning, depression, fatigue, pain, dyspnea, insomnia, appetite loss, and financial difficulties. On average, patients with breast cancer report lower physical and cognitive functioning, more depression, fatigue, dyspnea, and insomnia than patients with prostate cancer (Tables 3 and 4). Compared to patients with colorectal cancer, they report lower cognitive functioning, more pain, and insomnia. In contrast, patients with colorectal cancer report more diarrhea than patients with breast and prostate cancer. They also report more financial difficulties than patients with prostate cancer. Regarding appetite loss, the post-hoc comparisons reveal no significant differences between patient groups.

**Table 3:** Results of 3 (tumor site) × 3 (time of measurement) repeated measures ANCOVAs of EORTC QLQ C-30 functioning scales, depression, and fear of progression (240 ≤ *n* ≤ 282)

Scale	Site	Means (M), Standard Deviations (SD) or Standard Errors (SE), resp., and Reference Values (REF)								
		M_1_	SD_1_	M_2_	SD_2_	M_3_	SD_3_	M_1,2,3_	SE(M_1,2,3_)	M_REF_
Physical functioning	Breast	65.57	19.78	73.95	17.71	72.72	19.30	**70.61**	1.98	
	Colorectal	70.25	18.15	77.92	16.23	79.14	18.39	**75.31**	1.60	
	Prostate	74.95	19.07	77.68	17.69	79.86	22.88	**78.14**	1.76	
	All	***70.52***^***a***^	19.22	***76.73***	17.18	***77.58***	20.41			**78.9**
Role functioning	Breast	50.21	28.93	63.92	27.66	60.76	29.60	**60.42**	2.76	
	Colorectal	49.21	28.11	64.31	26.07	65.25	29.11	**59.26**	2.26	
	Prostate	51.95	32.41	67.20	28.55	72.70	29.62	**62.54**	2.47	
	All	***50.42***^***a***^	29.77	***65.17***^***a***^	27.32	***66.49***^***a***^	29.70			**77.7**
Emotional functioning	Breast	50.82	26.56	75.68	21.18	58.01	25.81	**62.22**	2.48	
	Colorectal	60.06	24.68	73.97	22.62	67.77	21.89	**66.82**	1.99	
	Prostate	62.46	26.21	75.00	21.78	70.85	25.04	**69.36**	2.18	
	All	***58.30***^***a***^	26.07	***74.79***^***a***^	21.88	***66.10***^***a***^	24.57			**79.1**
Cognitive functioning	Breast	58.76	28.01	69.23	28.56	65.38	28.01	**66.66**	2.71	
	Colorectal	72.07	27.23	77.93	25.36	78.55	22.91	**75.69**	2.17	
	Prostate	78.19	23.95	84.40	21.28	78.19	24.32	**79.01**	2.39	
	All	***70.42***^***a***^	27.41	***77.68***^***a***^	25.65	***74.76***^***a***^	25.48			**88.9**
Social functioning	Breast	53.85	30.85	70.09	28.22	66.03	27.58	**66.42**	2.73	
	Colorectal	55.81	29.60	72.78	25.42	72.02	28.32	**66.48**	2.18	
	Prostate	53.90	28.96	70.74	23.78	68.79	29.66	**62.36**	2.40	
	All	***54.63***^***a***^	29.65	***71.35***^***a***^	25.64	***69.28***^***a***^	28.58			**86.7**
Global health	Breast	53.84	18.63	66.89	18.66	64.36	19.13	**62.72**	1.90	
	Colorectal	54.63	19.21	65.28	17.48	65.12	20.23	**61.23**	1.50	
	Prostate	53.23	20.71	64.96	16.51	63.89	22.47	**60.37**	1.66	
	All	***53.94***^***a***^	19.51	***65.61***	17.45	***64.50***	20.65			**64.3**
Depression	Breast	8.48	4.31	4.89	3.51	6.67	4.49	**6.50**	.45	
	Colorectal	6.06	4.18	4.63	3.58	5.44	3.95	**5.40**	.36	
	Prostate	5.51	4.16	4.01	4.08	4.71	4.47	**4.86**	.41	
	All	***6.55***^***a***^	4.37	***4.50***^***a***^	3.74	***5.54***^***a***^	4.33			**3.2**
Fear of progression	Breast	32.83	7.65	31.61	7.69	32.10	8.43	**31.74**	.95	
	Colorectal	32.63	8.58	32.39	7.77	31.55	8.85	**32.24**	.80	
	Prostate	30.16	7.80	30.11	8.34	29.76	8.84	**30.38**	.92	
	All	***31.92***^***a***^	8.12	***31.44***^***a***^	7.95	***31.15***^***a***^	8.74			**34.0**

^a^Significant (*p* ≤ .001) difference of a respective overall sample mean from the age-matched reference value of the German general population for the QLQ-C30 [23], the PHQ-9 [24], or the cutoff for dysfunctional FoP [32], respectively

**Table 4:** Results of 3 (tumor site) × 3 (time of measurement) repeated measures ANCOVAs of EORTC QLQ C-30 symptom scales (273 ≤ *n* ≤ 287)

Scale	Site	Means (M), Standard Deviations (SD) or Standard Errors (SE), resp., and Reference Values (REF)								
		M_1_	SD_1_	M_2_	SD_2_	M_3_	SD_3_	M_1,2,3_	SE(M_1,2,3_)	M_REF_
Fatigue	Breast	59.56	25.69	45.50	23.79	47.96	28.94	**49.07**	2.58	
	Colorectal	50.75	25.05	36.74	21.70	37.64	23.21	**42.77**	2.06	
	Prostate	43.01	26.47	33.05	24.19	32.13	29.11	**36.43**	2.25	
	All	***50.56***^***a***^	26.42	***37.90***^***a***^	23.59	***38.62***^***a***^	27.55			**29.2**
Nausea and vomiting	Breast	5.06	10.46	2.95	8.34	5.27	14.26	**3.36**	1.12	
	Colorectal	6.64	14.22	4.32	13.16	4.48	12.61	**5.42**	.91	
	Prostate	2.98	8.76	2.28	7.92	4.39	14.22	**3.79**	.99	
	All	***4.96***	11.64	***3.25***	10.35	***4.67***	13.59			**4.0**
Pain	Breast	39.61	30.23	30.74	26.77	35.93	32.00	**33.85**	2.87	
	Colorectal	27.33	29.11	19.52	25.11	20.12	25.04	**23.33**	2.27	
	Prostate	33.16	30.09	21.13	24.94	20.79	30.05	**25.13**	2.48	
	All	***32.63***	30.05	***23.10***^***a***^	25.86	***24.62***^***a***^	29.49			**32.8**
Dyspnea	Breast	46.75	29.25	33.77	24.48	31.17	31.69	**36.71**	2.86	
	Colorectal	30.86	29.43	27.78	27.53	24.69	29.31	**28.34**	2.30	
	Prostate	21.99	27.46	20.57	28.96	21.28	27.59	**21.05**	2.52	
	All	***32.26***^***a***^	30.23	***27.00***	27.62	***25.33***	29.58			**23.2**
Insomnia	Breast	57.81	35.29	49.79	34.95	45.57	34.66	**50.99**	3.40	
	Colorectal	45.60	31.98	37.74	28.39	38.68	32.90	**40.07**	2.78	
	Prostate	43.26	37.81	32.62	33.15	34.40	34.72	**37.50**	3.04	
	All	***48.27***^***a***^	35.37	***39.43***^***a***^	32.59	***39.19***^***a***^	34.19			**30.2**
Appetite loss	Breast	12.24	22.76	5.06	16.09	10.13	22.24	**8.76**	2.28	
	Colorectal	21.91	31.63	11.73	23.82	12.35	22.60	**15.58**	1.85	
	Prostate	10.75	20.94	7.17	20.17	11.47	23.31	**9.84**	2.05	
	All	***15.48***^***a***^	26.45	***8.33***	20.79	***11.43***	22.67			**8.2**
Constipation	Breast	14.72	28.35	12.99	24.28	15.15	25.68	**13.81**	2.54	
	Colorectal	16.98	28.28	12.65	23.56	18.52	28.58	**17.16**	2.03	
	Prostate	21.63	27.96	14.89	26.15	15.96	27.53	**16.61**	2.23	
	All	***17.92***^***a***^	28.23	***13.50***^***a***^	24.59	***16.73***^***a***^	27.39			**8.2**
Diarrhea	Breast	11.40	21.47	5.70	14.80	8.77	20.63	**7.18**	2.64	
	Colorectal	32.08	35.61	23.27	29.52	31.13	35.71	**29.16**	2.12	
	Prostate	12.09	23.58	7.69	17.97	13.55	25.33	**11.93**	2.34	
	All	***19.66***^***a***^	29.88	***13.19***^***a***^	23.85	***19.05***^***a***^	30.31			**7.4**
Financial difficulties	Breast	27.78	32.42	24.79	30.60	27.35	35.53	**23.22**	3.28	
	Colorectal	31.80	36.40	29.66	35.24	26.30	33.97	**28.88**	2.61	
	Prostate	11.96	25.48	12.68	25.60	10.51	23.66	**15.06**	2.91	
	All	***24.13***^***a***^	33.08	***22.70***^***a***^	31.77	***21.39***^***a***^	32.25			**9.5**

^a^Significant (*p* ≤ .001) difference of a respective overall sample mean from the age-matched reference value of the German general population for the QLQ-C30 [23]

Effects of time of measurement are significant and mostly at least moderate (*η*^*2*^ ≥ 0.06) on all but three outcomes (fear of progression, nausea and vomiting, and financial difficulties). They primarily indicate improvement during rehabilitation that is stable at 9 months (Tables 3 and 4). Regarding emotional and cognitive functioning, depression, appetite loss, and diarrhea, there also is some deterioration at 9 months, however.

Finally, there are some significant and generally small interaction effects between tumor site and time of measurement indicating differential change across time (Tables 2, 3, 4). Compared to patients with colorectal or prostate cancer, patients with breast cancer improve to a greater extent during rehabilitation in emotional functioning but deteriorate again to a greater extent thereafter. Depression follows a similar pattern. Regarding dyspnea, patients with breast cancer show greater improvement during rehabilitation and thereafter appear to be stable. Patients with colorectal cancer show a comparable change regarding appetite loss.

### Comparison with reference values

Compared to EORTC QLQ-C30 norms from the German general population [30] (see last column of Tables 3 and 4), the present sample scores significantly lower on average on all EORTC QLQ-C30 functioning scales on nearly all time points except for physical functioning and the Global Health scale. The mean effect size (|*d*|= 0.74) is moderate (*d* > 0.50) at the beginning of rehabilitation, and smaller afterwards (mean |*d’s*| being 0.32 and 0.36, respectively, values not given in Table 3). Complementarily, the sample scores significantly higher throughout on 5 of the 9 symptom scales, whereby moderate to large effect sizes result in fatigue and insomnia (0.81 and 0.51, respectively, values not given in Table 4), which decrease thereafter. Similarly, the sample’s mean depression score at each time point is significantly higher than the suggested reference value [31] with moderate effect sizes at the beginning of rehabilitation and at follow-up. In contrast, FoP means are significantly lower than the cutoff for dysfunctional FoP [32].

## Discussion

Employing an RM-ANCOVA design that included comparable proportions of patients with breast, colorectal, and prostate cancer attending inpatient rehabilitation and the covariates patient age and TsD, this study investigated effects of tumor site, time of measurement, and their interaction on HRQoL, depression, and FoP. Given their effect sizes being small, even where significant, we found the influence of the covariates to be weak except for the association between higher patient age and lesser financial difficulties. The latter may in part reflect the sample composition, which included about 31% of retired participants living on regular pension. In addition, it should be noted that the relationship of patient age with social functioning and financial difficulties, respectively, is in line with previous research [1]. Regarding effects of tumor site, we found significant differences between patients with breast, colorectal, and prostate cancer in HRQoL and depression across time independent of patient age and TsD. Albeit mostly small in terms of effect size, these differences may be understood as partly reflecting persisting care needs specific to a particular group of patients. Primarily, this applies to patients with colorectal cancer who report a comparatively higher diarrhea symptom burden, thus reflecting the only larger effect (*η*^2^ ≥ 0.14) of tumor site. It also applies to patients with breast cancer, however, who show greater impairment on the EORTC QLQ-C30 scales of physical and cognitive functioning as well as on six of the symptom scales and on PHQ-9 depression. These differences may at least in part be due to the effects of prior treatment under acute care or surgery. In particular, receiving endocrine therapy, e.g., might have contributed to breast cancer patients’ higher levels of functional impairment or symptoms. Unfortunately, subsample sizes of breast cancer patients not having received, having completed, or still receiving endocrine therapy, respectively, did not allow valid testing of this hypothesis here. Therefore, further research aimed at explaining such differences between tumor diagnostic groups is required, as these thus far have often been covered merely descriptively by previous studies [24, 29].

The significant and in terms of effect size moderate to large changes in most HRQoL domains and depression during oncological inpatient rehabilitation and beyond may be seen as the main result of the present study. As to be expected from previous research, these changes indicate general improvement during rehabilitation. These are most prominent in role, emotional, and social functioning, fatigue, and depression. However, while improvement in most of these outcomes remained stable 9 months after rehabilitation, there was significant deterioration in some others, which in part also appeared to apply only to subgroups of patients, like, e.g., emotional functioning in patients with breast cancer or diarrhea in patients with colorectal cancer. Here again, the question of how to explain these differential changes arises. In the present study, ex-post analyses suggested that compared to colorectal or prostate cancer patients a higher proportion of patients with breast cancer indicated having experienced “distressing life events” (without further specification) during the previous 6 months (51.2%, 43.6%, and 27.1%, respectively, *p* < 0.005) and hospital re-admissions related to their disease and surgery (25%, 11%, and 7%, respectively, *p* < 0.05). Of course, this may not generalize beyond the current sample. However, as some previous studies have in part observed indications of differential deterioration in HRQoL after inpatient rehabilitation [23, 24], too, further research on possible determinants of such trajectories is again called for.

Comparing the overall sample means of each measurement point to available reference values for HRQoL, depression, and FoP revealed that in spite of obvious improvement during rehabilitation, patients continued to score significantly lower than available reference values in some of these outcomes. This is in line with the results of earlier studies [23, 25]. Although effect sizes for differences had decreased at follow-up, moderate ones were still observed there for emotional, cognitive, and social functioning. Regarding FoP, the sample’s mean scores were significantly lower than the cutoff suggested for dysfunctional FoP [32] from the first measurement point onwards. This might suggest the majority of the sample had no need for treatment in this respect, thus leading to no detectable improvement in FoP in contrast to other studies [19, 21]. In fact, the latter of these studies reports post-and follow-up FoP mean scores of about *M* = 32 that are only slightly higher than those reported here.

Aside from providing suggestions for further research, the results of this study may inform the practice of oncological rehabilitation and follow-up care. Implementing screening for distress in its various forms at the beginning of rehabilitation, e.g., may help tailor rehabilitative treatments even better to the individual patient’s needs and thus achieve a major principle of rehabilitative services. When compared to available norms for the German general population, patients scored still notably worse in some HRQoL domains and symptoms and higher on depression at the end of rehabilitation or at the 9-month follow-up in spite of prior improvement. This obviously suggests the need for additional follow-up care. The deterioration observed in breast cancer patients, e.g., in emotional functioning at follow-up, points in the same direction. The task of providing appropriate follow-up care would imply integrating the detection of respective care needs and the provision of appropriate interventions. This might be accomplished through currently discussed national survivorship programs that integrate a wide range of medical and psychosocial services and the collaboration of acute care, in- and outpatient rehabilitation, and cancer counseling centers [39–42].

## Strength and limitations

Strengths of this study include its multi-factorial and multi-center approach, the inclusion of three major tumor entities to comparable proportions, the comparatively large sample size, and a follow-up interval covering 9 months. However, some limitations also need to be considered. First, the longitudinal observational design precludes causal inferences on rehabilitation effects. Second, in spite of the largely positive results of the dropout analyses, selection effects cannot completely be excluded as the response rate at study entry could not be recorded. Third, the final sample size is somewhat lower than originally planned, so some other than main effects may have been missed. Fourth, gender and tumor site are in part confounded. Fifth, generalizing findings to other tumor entities and other than inpatient settings is not possible, thus indicating the need for further research.

## Conclusion

Oncological inpatient rehabilitation has the potential to significantly improve patients’ HRQoL and depression. Some HRQoL functions and symptoms may deteriorate again somewhat later, indicating the need for subsequent follow-up care that can be addressed through survivorship programs. Research addressing the possible differential change of HRQoL outcomes after rehabilitation and its determinants might help improve rehabilitation and survivorship services further.

## Data Availability

The data on which the present analyses are based will be available from the corresponding author upon reasonable request.
